# Cryptotanshinone-Induced Permeabilization of Model Phospholipid Membranes: A Biophysical Study

**DOI:** 10.3390/membranes14060118

**Published:** 2024-05-21

**Authors:** Julia Ortiz, Francisco J. Aranda, José A. Teruel, Antonio Ortiz

**Affiliations:** Departamento de Bioquímica y Biología Molecular-A, Facultad de Veterinaria, Campus de Espinardo, Universidad de Murcia, E-30100 Murcia, Spain; julia.ortiz@um.es (J.O.); fjam@um.es (F.J.A.); teruel@um.es (J.A.T.)

**Keywords:** cryptotanshinone, phospholipid membranes, membrane permeabilization, molecular dynamics

## Abstract

The Danshen terpenoid cryptotanshinone (CPT) is gaining enormous interest in light of its various outstanding biological activities. Among those, CPT has been shown to interact with cell membranes and, for instance, to have antibacterial activity. Several works have shown that CPT alone, or in combination with other drugs, can effectively act as an antibiotic against various infectious bacteria. Some authors have related the mechanism underlying this action to CPT–membrane interaction. This work shows that CPT readily partitions into phosphatidylcholine membranes, but there is a limiting capacity of accommodation of ca. 1 mol CPT to 3 mol phospholipid. The addition of CPT to unilamellar liposomes composed of 1-palmitoyl-2-oleoylphosphatidylcholine (POPC) causes membrane permeabilization, as shown by fluorescent probe leakage. This process has been kinetically studied, as well as its modulation by incorporation of phosphatidylethanolamine or phosphatidylglycerol, as a model for pathogenic cell membranes. The thermotropic behavior of 1,2-dimyristoylphosphatidylcholine (DMPC) model membranes is weakly affected by CPT, but the terpenoid causes significant dehydration of the polar region of the bilayer and weak disordering of the acyl chain palisade, as observed in Fourier-transform infrared spectroscopy (FTIR) results. Small-angle X-ray scattering (SAXS) shows that CPT increases DMPC bilayer thickness, which could be due to localization near the phospholipid/water interface. Molecular dynamics (MD) simulations show that the lateral diffusion coefficient of the phospholipid increases with the presence of CPT. CPT extends from the polar head region to the center of the bilayer, being centered between the carbonyl groups and the unsaturated region of the POPC, where there is greater overlap. Interestingly, the free energy profiles of a water molecule crossing the lipid membrane show that the POPC membrane becomes more permeable in the presence of CPT. In summary, our results show that CPT perturbs the physicochemical properties of the phospholipid membrane and compromises its barrier function, which could be of relevance to explain part of its antimicrobial or anticancer activities.

## 1. Introduction

**Figure 1:**
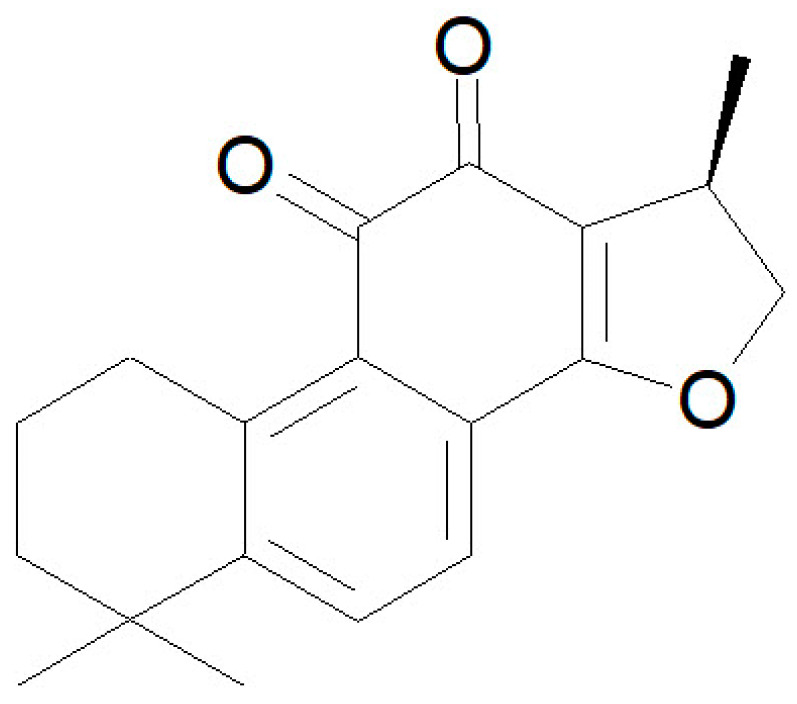
The chemical structure of cryptotanshinone.

Tanshinones constitute one of the main components, in terms of their biological and therapeutic activities, of *Salvia miltiorrhiza*, whose dried roots are known as Danshen [1]. Danshen has been used in traditional Chinese medicine for centuries, and it contains biologically active hydrophilic and hydrophobic compounds, tanshinones, constituting the major lipophilic components [2]. Tanshinones are diterpene compounds involving more than forty specimens to date [3], among which cryptotanshinone (CPT) can be cited as one of the principals, based on its biological activities. CPT is a diterpene quinone (Figure 1) that can permeate cells and present numerous pharmacological activities, particularly for the treatment of cancer and cardiovascular diseases [4]. The relevance of this biologically active compound has been highlighted in a number of excellent reviews [4,5,6,7]. There are multiple pieces of evidence on the efficacy of CPT for the treatment of cancer [5,8], among which we can recently cite its use in the targeting of mutations in colorectal cancer [9], or as a codrug for the treatment of osteosarcoma [10]. Other interesting activities include anti-inflammatory, anti-fibrotic, or neuroprotective effects [6].

The potential molecular mechanisms underlying this vast number of pharmacological and biological actions of CPT often involve different cell signaling pathways that have been recently reviewed [4,6]. In addition to the above-mentioned activities, CPT has been shown to present antibacterial activity [11,12,13,14] whose molecular mechanism could be completely different, being the result of its interaction with the cell membrane, resulting in membrane damage and subsequent cell death [11,12]. However, to date, there are no published results on the interaction of CPT with biological membranes, and the modulation of the structure and function of phospholipid bilayers. Hence, to investigate the effect of the terpenoid on the structure and function of phospholipid membrane bilayers, this work presents an experimental and MD study on the interaction of CPT with model phospholipid vesicles of various compositions, using appropriate physical techniques and permeability assays. The provided results show the potential preferent location of CPT within the bilayer, and how it can lead to membrane alteration and permeabilization, resulting in aqueous contents leakage.

## 2. Materials and Methods

### 2.1. Materials

1-Palmitoyl-2-oleoyl-*sn*-glycero-3-phosphocholine (POPC), 1-palmitoyl-2-oleoyl-*sn*-glycero-3-phosphoethanolamine (POPE), 1-palmitoyl-2-oleoyl-*sn*-glycero-3-phospho-glycerol (POPG), 1,2-dimyristoyl-*sn*-glycero-3-phosphocholine (DMPC), and 1,2-dipalmitoyl-*sn*-glycero-3-phosphocholine (DPPC) were purchased from Avanti Polar Lipids Inc. (Birmingham, AL, USA). Cryptotanshinone (CPT) was purchased from Cymit-Química (Barcelona, Spain). 5(6)-carboxyfluorescein (CF) (99% by HPLC) was from Sigma-Aldrich (Madrid, Spain). All the other reagents were of the highest purity available. The inorganic salts and buffers were of analytical grade. Purified water was deionized in Milli-Q equipment from Millipore (Millipore, Bedford, MA, USA) up to a resistivity of ca. 18 MΩ. Stock solutions of the various phospholipids and CPT were prepared in chloroform and stored at −80 °C. Phospholipid phosphorus was determined according to the method of Böttcher [15]. The buffer used throughout the work was 150 mM NaCl and 5 mM Hepes pH 7.4 unless otherwise stated. Water and all the buffer solutions used in this work were filtered through 0.2 μm filters before use. The osmolarity of all the buffers and solutions was checked using an Osmomat 030 osmometer (Gonotec, Berlin, Germany).

### 2.2. Partition of CPT into Phospholipid Bilayers

To investigate the partitioning of CPT into phospholipid membranes, POPC was conveniently used since this phospholipid is in the fluid state at room temperature. Mixtures of 2 µmol of POPC and various increasing amounts of CPT were prepared essentially as described below for DSC. Multilamellar liposomes were obtained by adding 1 mL of the above-mentioned buffer and vortexing. These liposomes were left to rest at room temperature for 3 h to allow the sedimentation of any CPT insoluble crystals. A small aliquot of the supernatants (free of CPT crystals, as observed by light microscopy) was carefully collected, and the concentration of phospholipid and CPT was determined. CPT was determined by absorbance at 450 nm after the addition of absolute ethanol (4:1 by volume, ethanol/sample ratio). The molar extinction coefficient of CPT at 450 nm, determined in that same solvent mixture, was ɛ = 2.86 mM^−1^ cm^−1^.

### 2.3. Vesicle Contents Release

CPT-induced vesicle contents release was monitored with the CF assay, for which CF was entrapped within the phospholipid unilamellar vesicles of the indicated composition, and its leakage was followed by the increase in fluorescence due to dilution of the probe to the external medium [16]. Multilamellar vesicles were prepared by the dry film hydration method, by vortexing 5 μmol of POPC with 0.5 mL of a buffer containing 50 mM CF and 10 mM Hepes, pH 7.4, at room temperature, and large unilamellar vesicles were obtained by 21 times extrusion of the multilamellar vesicles through two stacked polycarbonate filters (0.1 μm pore diameter). Sephadex G-50 gel filtration, with 100 mM NaCl and 10 mM Hepes pH 7.4 as elution buffer, was used to separate the vesicles from non-encapsulated CF. CPT was added at the indicated concentrations from stock solutions in DMSO, and it was confirmed that the same volumes of pure DMSO did not induce any leakage. Maximum leakage (100%) was established by disrupting the liposomes with 5% Triton X100, which provoked the complete release of CF to the external medium. The percentage of CF leakage was calculated as:

$$\%\;CF\;Leakage=\frac{\left(F_{t}-F_{i}\right)\cdot 100}{F_{d}-F_{i}}$$

where F_t_ is the fluorescence at a given time after CPT injection, F_i_ is the initial fluorescence, and F_d_ is the maximum fluorescence obtained after addition of the detergent Triton X100.

### 2.4. Differential Scanning Calorimetry

Multilamellar vesicles for DSC measurements were prepared by the dry film hydration method. Briefly, 2 µmol of DMPC or DPPC and CPT as indicated were mixed in chloroform, and the organic solvent was gently evaporated using dry N_2_ to obtain a thin film at the bottom of a glass tube. The last traces of solvent were removed by a further 3 h desiccation under a high vacuum. To the dry samples, 2 mL of a buffer containing 100 mM NaCl, 0.1 mM EDTA, and 10 mM Hepes pH 7.4 was added, and vesicles were formed by vortexing the mixture at 50 °C. The experiments were performed using a MicroCal MC2 calorimeter (MicroCal, Northampton, NC, USA) at 1 mM phospholipid concentration and 60 °C h^−1^ heating scan rate. Three consecutive heating scans were carried out for each sample, the last one being taken for analysis.

### 2.5. Fourier-Transform Infrared Spectroscopy

Multilamellar vesicles for Fourier-transform infrared spectroscopy (FTIR) were prepared by the dry film hydration method, in 40 μL of the same buffer described above prepared with D_2_O. The samples were placed between two CaF_2_ windows (25 × 2 mm) separated by 25 μm Teflon spacers and mounted onto a Symta cell mount. Infrared spectra were acquired in a Nicolet 6700 Fourier-transform infrared spectrometer (Madison, WI, USA). Each spectrum was obtained by collecting 64 interferograms with a nominal resolution of 2 cm^−1^. The equipment was continuously purged with dry air to minimize the contribution peaks of atmospheric water vapor, and the sample holder was thermostated using a Peltier device (Nicolet Proteus System). Spectra were collected at 2 °C intervals, allowing 5 min equilibration between temperatures. The D_2_O buffer spectra taken at the same temperatures were subtracted interactively using either Omnic (8.0.342) or Grams (7.02) (Galactic Industries, Salem, NH, USA) software.

### 2.6. X-ray Scattering

Samples for X-ray scattering analysis were prepared essentially as described above for FTIR, in 1 mL of 100 mM NaCl, 0.1 mM EDTA, and 10 mM Hepes pH 7.4 at 40 °C. The samples were centrifuged in a bench microfuge, and the pellets were placed in the sample holder of the diffractometer with the aid of a spatula. A steel holder with cellophane windows was used, providing good thermal contact to the Peltier heating unit. Typical exposure times were 5 min, leaving a 10 min equilibration period prior to each measurement. Small angle (SAXS) X-ray scattering data were collected using a Kratky compact camera (M. Braun-Graz Optical Systems, Graz, Austria), equipped with a linear position sensitive detector (PSD; M. Braun, Garching, Germany), monitoring the s range (s = 2 sin θ/λ, 2θ = scattering angle, λ = 1.54 Å) between 0.0075 and 0.07 Å^−1^. Nickel-filtered Cu K_α_ X-rays were generated by a Philips PW3830 X-ray generator (Eindhoven, The Netherlands) at 50 kV and 30 mA. The calibration of the detector position was performed by using silver stearate (d-spacing at 48.8 Å) as the reference material.

### 2.7. Molecular Dynamics Simulations

The 3D molecular structure of CPT was obtained from the PubChem Substance and Compound databases [17] through the unique chemical structure identifier CID 160254. All the MD simulations were conducted using GROMACS 5.0.7 and 2018.1 [18]. The CHARMM36 force field parameters for DMPC, POPC, CPT, water, Cl^−^, and Na^+^ were obtained from CHARMM-GUI [19,20,21]. The membrane bilayers were formed by 2 leaflets oriented normal to the z-axis with a total of 128 molecules of DMPC or POPC with and without 14 molecules of CPT, and a water layer containing a total of 6400 water molecules (TIP3 model), 12 Na^+^, and 12 Cl^−^. The initial membrane structures were built with the aid of Packmol software (v 20.14.4) [22].

All the systems were simulated using the NpT-ensemble at 312 K for DMPC membranes and at 298 K for POPC membranes. The pressure was controlled semi-isotropically at a pressure of 1 bar and compressibility of 4.5 × 10^−5^ bar^−1^. The cutoffs for van der Waals and short-range electrostatic interactions were 1.2 nm, and a force-switching function was applied between 1.0 and 1.2 nm [23]. The simulations were initiated by a 20 ns run, using the V-rescale thermostat and the Berendsen barostat [24], followed by a 200 ns run using the Nosé–Hoover thermostat [25] and the Parrinello–Rahman barostat [26]. Graphical representations were conducted with PyMOL 2.3.0 [27]. Analyses of the trajectories were conducted over the last 60 ns using the Gromacs tools.

Area per lipid was calculated from the lateral dimensions of the Malone simulator box (the area of the xy plane) divided by the number of lipids in each leaflet. The diffusion constants (D) were calculated from the slope of the mean square displacement (MSD) versus time using the Einstein relation:

$$D=\underset{\Delta t\to \infty }{\lim}\frac{{❬\left|\Delta \bar{r}\right|❭}_{t_{0}}^{2}}{2d\Delta t}$$

where MSD = ${❬{\left|∆\bar{r}\right|}^{2}❭}_{t_{0}}$, and d is the dimensions (e.g., d = 2 for lateral diffusion). The deuterium order parameter across the acyl chain, S_CD_, is defined as:

$$S_{\mathrm{CD}}=\frac{3}{2}❬\cos^{2}\theta ❭-\frac{1}{2}$$

where θ is the angle between a CD bond and the bilayer normal. A value of −0.5 indicates a perfectly ordered acyl chain in an all-trans conformation [28]. The membrane thickness was computed by calculating the phosphorous atom’s distance between both leaflets. The cluster size distribution of CPT in the membranes was calculated as the number of CPT molecules found within 0.30 nm of the analyzed trajectory.

The free energy profile of an H_2_O molecule crossing the POPC bilayer, in the absence and the presence of CPT molecules, was calculated using the potential of mean force (PMF) procedure [29]. The starting configurations were the final configurations obtained in the MD simulations. An H_2_O molecule from the water phase was pulled to the lipid bilayer center along the *z*-axis using the umbrella method [30]. A harmonic restraint of 1000 kJ mol^−1^ nm^−2^ and a pulling rate of 0.007 nm ps^−1^ were applied to distance z between the center of mass of the H_2_O molecule and the POPC bilayer. This procedure was repeated three times with different water molecules without altering the membrane structure. The H_2_O molecule configuration was then sampled and constrained at different z distances, allowing free motion in the xy plane. The free energy profile was calculated by using the weighted histogram analysis method [31] included in GROMACS tools. The calculated free energy profile was considered symmetric across the POPC bilayer center.

## 3. Results and Discussion

CPT, a diterpene quinone, can be considered a phenanthraquinone derivative (Figure 1); it is an essentially water-insoluble compound but is soluble in most organic solvents. This highly hydrophobic character should make CPT very prompt to partition into phospholipid membranes and, in fact, CPT has been described as a cell-permeable compound [8]. In this work, we carried out an experimental MD approach to study the incorporation of CPT into model phospholipid membranes, and its effect on the structure and function of model phospholipid membranes of various compositions, to obtain information on CPT–phospholipid interactions at the molecular level. Up to five different phospholipid species were used. To study thermotropic transitions, multilamellar vesicles made of DMPC or DPPC were used, since these phospholipids present thermal transitions in a temperature range appropriate for this type of study. These systems were analyzed using DSC, FTIR, and SARS techniques. On the other hand, for the study of CPT-induced membrane permeabilization, through CF fluorescence, POPC, POPG, and POPE were used, since these phospholipids, containing an oleic acid at the *sn*-2 position of glycerol, form fluid bilayers above 0 °C.

### 3.1. Partition of CPT into POPC Membranes

Over the course of the study, it was observed that upon the formation of CPT/POPC liposomes, a fraction of the diterpene did not incorporate into the vesicles and appeared in the form of orange crystals, as observed by light microscopy. Since CPT is essentially water-insoluble, it was concluded that the fraction of terpenoid non-incorporated into the membrane readily crystallized.

**Figure 2:**
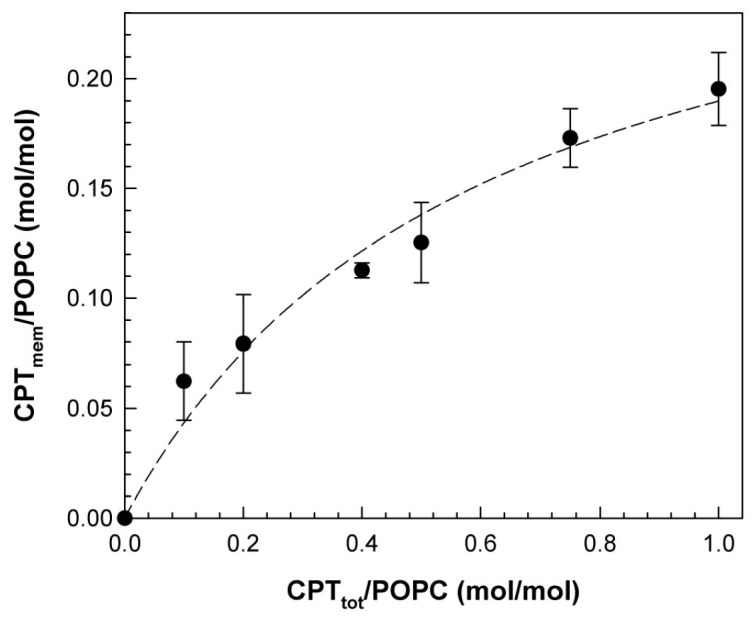
Partition of CPT into POPC membranes. The actual molar ratio of CPT in the membrane (CPT_mem_/POPC), determined as described in Materials and Methods, is plotted against the total initial CPT to POPC molar ratio (CPT_tot_/POPC). Data correspond to the mean of three independent experiments ± SD (error bars).

This finding led to the need to determine the actual concentration of CPT incorporated into the membrane. Mixtures of POPC and CPT at various initial proportions were prepared, and the incorporated CPT was determined (Figure 2). The actual CPT/POPC membrane molar ratio showed a hyperbolic dependence with the total initial concentration of CPT. Thus, the data shown in Figure 2 were adjusted to the following equation:

$$R_{mem}=\frac{R_{sat}\cdot \frac{CPT_{tot}}{POPC}}{R_{50}+\frac{CPT_{tot}}{POPC}}$$

where R_mem_ is the molar ratio of CPT in the membrane to total POPC, R_sat_ is the saturation membrane molar ratio of CPT, R_50_ is the CPT concentration necessary to reach 50% of R_sat_, and CPT_tot_ is the total CPT concentration. This fitting yielded a value for R_sat_ of 0.30 ± 0.04 and R_50_ of 1.2 ± 0.3. These interesting results indicated that the fluid membrane of POPC had a limiting capacity on incorporating CPT, corresponding to a CPT_mem_/POPC molar ratio of ca. 0.33 (the non-incorporated diterpene crystallized in the form of insoluble crystals, as commented above). According to these results, it was clear that POPC membranes had a limited capacity to accommodate CPT, which was around 1 mol CPT to 3 mol phospholipid. This conclusion can be extrapolated to any phosphatidylcholine membrane, since POPC is widely accepted and used as an average representative for fluid phosphatidylcholine membranes [32] and, in particular, for membrane permeability studies [33]. Therefore, it is particularly important to take this fact into consideration when the membrane-related effects of CPT are evaluated, since the actual concentration of this bioactive compound in the membrane is significantly smaller than that added.

### 3.2. CPT-Induced Membrane Permeabilization

**Figure 3:**
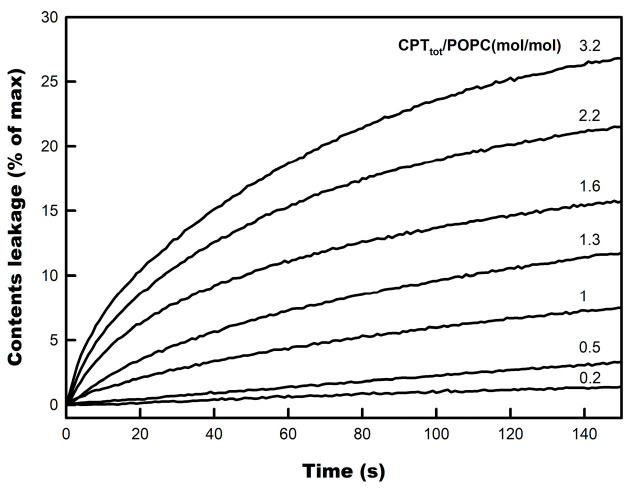
Content leakage curves for CPT-induced permeabilization of POPC LUV. The concentration of POPC was kept constant at 20 µM. Numbers on the curves indicate the molar ratio of total CPT to phospholipid in the cuvette (CPT_tot_/POPC).

Figure 3 shows the time course curves of CPT-induced content leakage of POPC large unilamellar vesicles (LUV). Increasing concentrations of CPT were added from outside, as a DMSO solution, to pre-formed vesicles at a constant concentration, and the leakage of CF was monitored by the concomitant increase in probe fluorescence. Concentrations of the diterpenoid as low as 4 µM (CPT_tot_/phospholipid molar ratio = 0.2) already gave rise to a slow but measurable liposome content leakage to the external medium, which progressed to reach 100% if allowed to continue.

The rate and extent of CPT-induced leakage continued progressively increasing as its concentration was raised above that ratio, indicating that the R_sat_ value was not reached. These results indicate that a fraction of added CPT rapidly incorporated into the POPC membranes and compromised their barrier function, resulting in increased permeability to water-soluble compounds, like CF.

**Figure 4:**
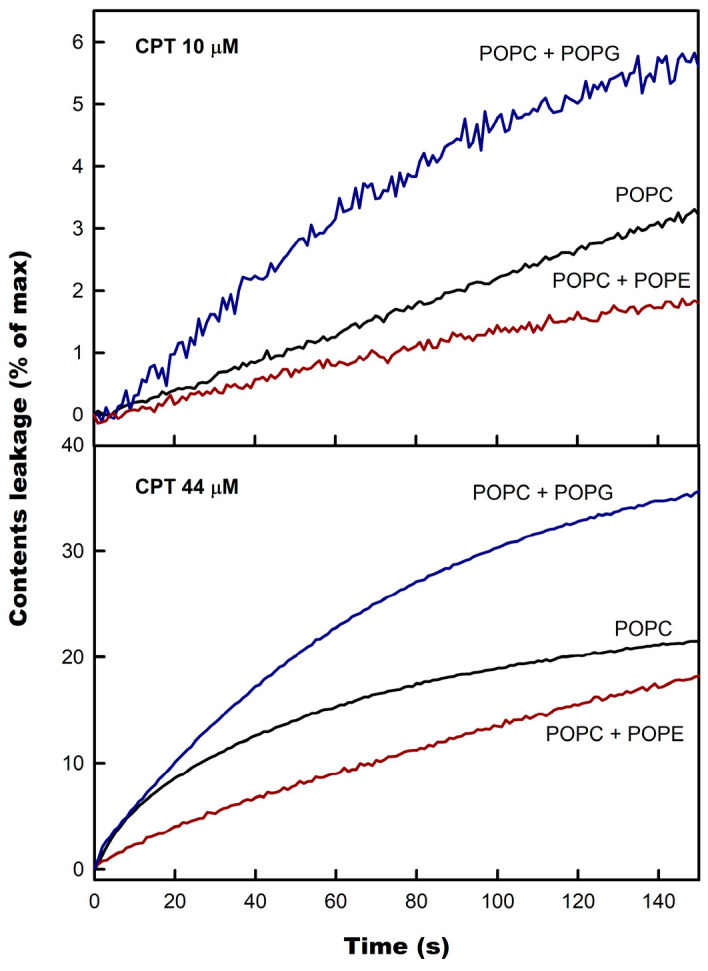
The influence of membrane composition on CPT-induced leakage. Experiments were conducted under the same conditions as in Figure 3, for liposomes composed of pure POPC (black), POPC/POPG (5:1.7, mol/mol) (blue), and POPC/POPE (5:1.7, mol/mol) (red), at two different CPT concentrations, as indicated.

The influence of phospholipid membrane composition on CPT-induced leakage was checked in liposomes containing an addition of either POPG or POPE (Figure 4). POPE was selected because of its small headgroup and rich lipid polymorphism [34], whereas POPG has an anionic character and a voluminous headgroup [35].

It was observed that, irrespective of CPT concentration, the presence of POPE protected the membrane against permeabilization, whereas POPG significantly enhanced CPT-induced vesicle content leakage. This effect was observed both at low and high CPT/phospholipid ratios, suggesting that it is the nature of the bilayer itself, and not specific phospholipid–CPT interactions that account for it. This is a very interesting result since it showed that target membrane composition determined the effect of CPT. It means that CPT membrane action is not indiscriminate, and it is expected to present different effects on different cells, as commented below.

The protective effect of phosphatidylethanolamine is not new, since this phospholipid has been reported to inhibit leakage in other systems [36,37,38,39,40]. Due to its small headgroup and the interlipid hydrogen bonding initiated by the amine group [41], phosphatidylethanolamine can increase membrane compactness and stabilize the surface state of the phospholipid membrane [36], which would impede the proper insertion of CPT necessary for permeabilization, thus reducing leakage.

The significant enhancement in CF leakage by POPG is of great importance, given the fact that both bacterial and eukaryotic cancer cells have negatively charged phospholipids in the outer monolayers [42,43,44]. Thus, it seems that negatively charged membranes, as a model of pathogenic membranes [45], are more prompt to permeabilization, which might contribute to explaining part of the anticancer and antimicrobial actions of CPT.

### 3.3. Modulation of DMPC Thermotropic Transitions by CPT

**Figure 5:**
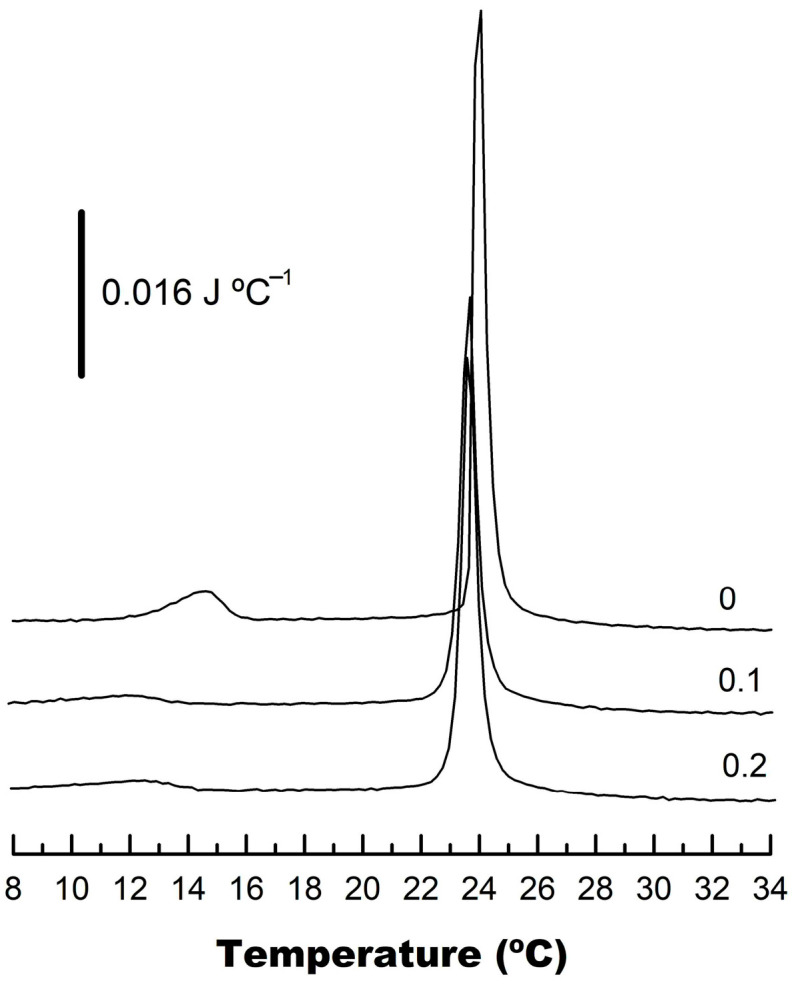
High-sensitivity DSC heating thermograms for mixtures of CPT with DMPC. CPTtot/DMPC molar ratios are indicated on the curves. Scans were carried out at 60 °C h^−1^.

To obtain information on CPT–phospholipid interactions, a saturated species of phosphatidylcholine, namely DMPC, was chosen. This phospholipid is appropriate for these types of studies since, upon heating, it displays the so-called pre-transition, from the gel L_β_ phase to the gel ripple P_β_′ phase, at around a T_c_ of 12.1 °C, and a main transition from the ripple P_β_′ phase to the liquid crystalline fluid, L_α_, phase around a T_c_ of 23.7 °C (Figure 5).

CPT was incorporated into DMPC liposomes by comixing prior to vesicle formation, at two different concentrations. It can be observed that the presence of CPT_tot_/DMPC 0.1 and 0.2, respectively, of the quinone had a significant effect on the pre-transition, which was shifted toward lower temperatures, from 12.1 to 9.3 and 8.0 °C, respectively, progressively widened, and decreased in area. However, the effect on the main gel-to-liquid crystalline phase transition was weak, the T_c_ shifting from 23.7 to 22.8 and 22.7 °C, 0.1 and 0.2 CPT_tot_/DMPC, respectively. In addition, the main transition peak was slightly widened. It was clear that the membrane-incorporated CPT did not establish sufficiently strong interactions with DMPC to significantly perturb its thermotropic behavior. Similar measurements were carried out using DPPC, and the results were qualitatively like those described above for DMPC. Since the pre-transition is mostly caused by the rearrangement of lipid headgroups, and the main transition by the melting of lipid acyl chains, our results showed a stronger effect of CPT at the level of the phospholipid polar region and a much smaller effect on lipid packing.

### 3.4. Effect of CPT on Acyl Chain and Polar Headgroup Regions of the DMPC Bilayer

**Figure 6:**
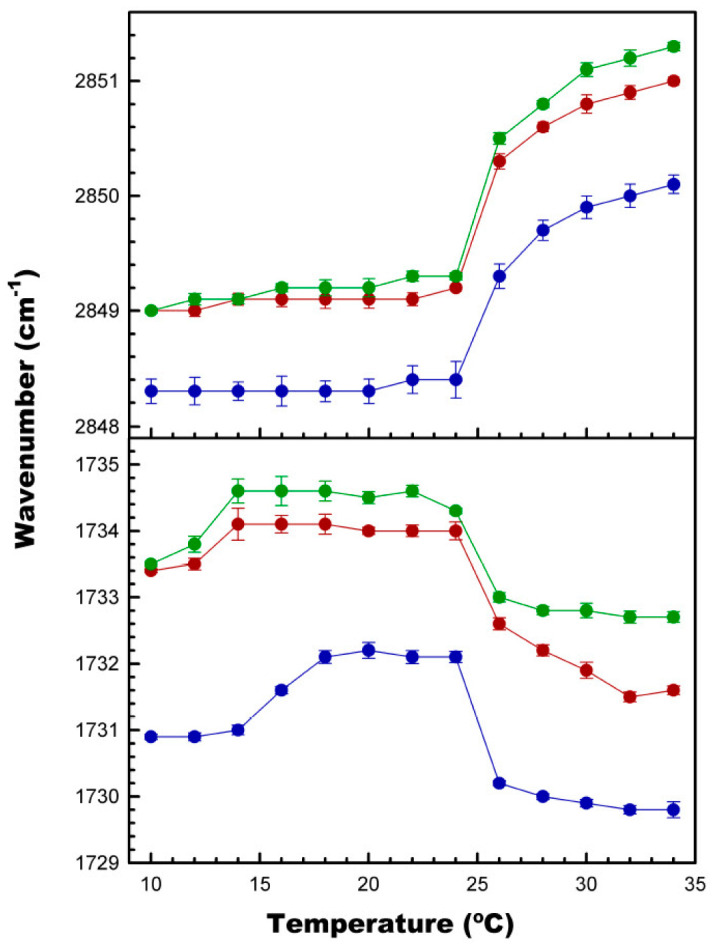
The effect of CPT on the region of the DMPC acyl chains and polar headgroups, determined by FTIR. Top: the effect on the maximum frequency of the ν_CH2_ symmetric stretching band. Bottom: the effect on the maximum frequency of the ν_CO_ stretching band. Plots correspond to pure DMPC (blue), and CPT_tot_/DMPC (mol/mol) 0.1 (red) and 0.25 (green). Spectra were collected from 10 to 34 °C, every 2 °C. Data correspond to the mean of three independent repetitions ± SD (error bars).

Using FTIR, it was possible to elucidate the potential effects of CPT on the two main moieties of the DMPC molecule: the acyl chain palisade and the lipid/water interface. Thus, the acyl chain region was studied by monitoring the frequency of the CH_2_ symmetric stretching band, ν_CH2_, and the polar region from the frequency of the C=O stretching band, ν_CO_ (Figure 6). The incorporation of 10 mol% of CPT into the DMPC resulted in a ~1 cm^−1^ shift of the maximum of the ν_CH2_ band towards higher values, in the whole temperature range. Further increasing the concentration of CPT to 20 mol% gave rise to an additional small increase of barely 0.3 cm^−1^, particularly in the fluid phase. These weak effects were in good agreement with the weak effect of CPT on the main gel-to-liquid crystalline phase transition of DMPC described above. In the case of the ν_CO_ band, the inclusion of 10 mol% of CPT shifted the maximum of the band about 2.4 cm^−1^ towards higher values, and, again, 20 mol% of the quinone just slightly enhanced this shift. This indicates that CPT was causing dehydration of the polar region of the membrane, in line with the effect of the terpenoid on the DMPC pre-transition shown above.

A similar study was conducted with liposomes composed of various phosphatidylcholines, and the main results are presented in Table 1. It was observed that in the case of DPPC, with longer acyl chains than DMPC, the effects were similar but significantly weaker. Furthermore, in the case of POPC, which is fluid at 25 °C, CPT also caused an additional shift in the maximum of both bands, as in the case of DMPC.

Taken together, the DSC and FTIR results clearly indicated that CPT did not significantly affect lipid packing, its effects being concentrated at the polar part of the bilayer.

### 3.5. Effect of CPT on DMPC Structural Parameters

**Figure 7:**
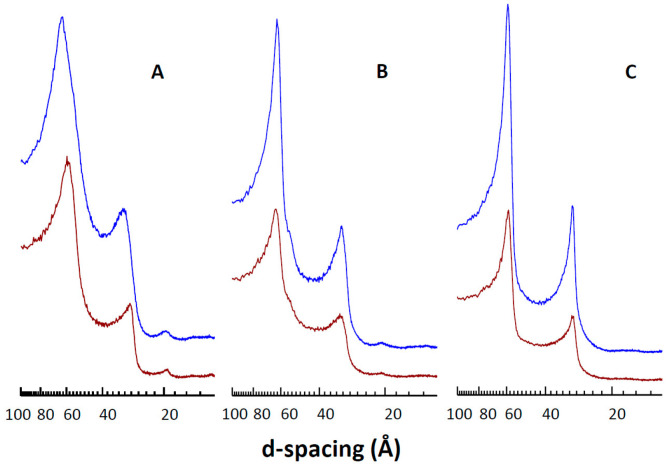
Effect of CPT on the SAXS profiles of DMPC. Diffractograms correspond to pure DMPC (red) and CPTtot/DMPC 0.1 (mol/mol) (blue), at 7 °C (**A**), 18 °C (**B**), and 34 °C (**C**).

SAXS was carried out to obtain information on the influence of CPT on the DMPC bilayers’ structural parameters. Measurements were carried out at three different temperatures corresponding to the L_β_ phase (7 °C), the gel ripple P_β_′ phase (18 °C), and the liquid crystalline L_α_ phase (34 °C) (Figure 7). Pure DMPC showed three reflections with relative distances at 1:1/1:2/1:3, corresponding to a multilamellar organization. The largest peak (the first-order reflection) gives the interlamellar repeat distance (d-spacing), a summation of the bilayer thickness, and the thickness of the water layer between bilayers [46]. The first-order reflection of pure DMPC appeared at a d-spacing of 59.6 Å at 7 °C, 64.6 Å at 18 °C, and 60.0 Å at 34 °C, in agreement with previous data [47]. The incorporation of 0.1 CPT_tot_/DMPC resulted in an increase in d-spacing below the main gel-to-liquid crystalline phase transition: 64.6 Å at 7 °C and 66.3 Å at 18 °C, whereas it did not change in the L_α_ phase: 60.5 Å at 34 °C. This widening effect could occur by an increase in the water layer between the DMPC membranes, or by a real increase in the bilayer thickness, due to the insertion of CPT.

### 3.6. Molecular Dynamics Simulations

**Figure 8:**
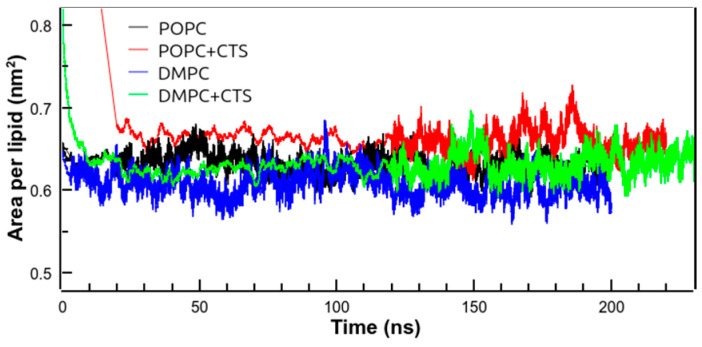
Area per lipid vs. simulated time for all simulated systems: POPC at 298 K (black), POPC + CPT at 298 K (red), DMPC at 312 K (blue), and DMPC + CPT at 312 K (green).

The area per lipid parameter is frequently used as a property of a lipid bilayer for validating MD simulations [48]. Figure 8 shows the time course of the area per lipid in different simulations.

It can be observed that the area per lipid remained mainly constant during the MD simulation time. The area per lipid of pure POPC and DMPC bilayers above the phase transition was 0.63 nm^2^ and 0.61 nm^2^, respectively (Table 2). Thus, our results are in good agreement with the reported data for POPC (0.61–0.64 nm^2^) [49,50] and for DMPC (0.61 nm^2^) [50,51]. In the presence of CPT, the area per lipid increased by around 5% in both membranes, probably due to the broadening produced solely by the presence of more non-phospholipid molecules in the membranes. Neither the order parameter (S_CD_) nor the thickness of the membranes changed significantly in the presence of CPT (Table 2), in total agreement with the SAXS data shown above.

In the presence of CPT, the lateral diffusion coefficient of the phospholipid increased by 41% and 57% in POPC and DMPC membranes, respectively (Table 2). This result indicated a higher molecular mobility in the membrane in the presence of CPT, which can result in local membrane destabilization (‘pores’ formation) and leakage. In this case, the term ‘pore’ within should be understood as zones in the membrane where membrane stability is decreased, resulting in membrane permeabilization [52].

**Figure 9:**
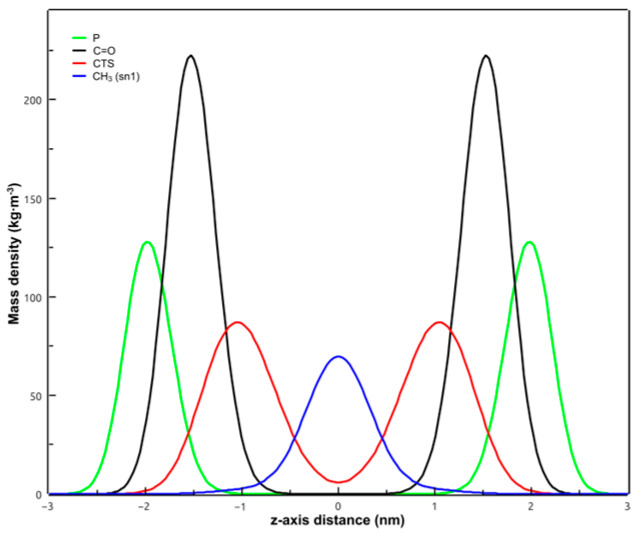
Mass density profiles along the z-axis of the simulation box of the simulated system of POPC + CPT. POPC phosphorus atoms are in green, CPT in red, POPC terminal methyl of *sn*-1 chains in blue, and POPC carbonyl groups in black. Curves are symmetrized around the center of the bilayer.

Figure 9 shows the mass density profile of the POPC membrane in the presence of CPT. Some important groups of the POPC molecule have been included: the polar head region (P atoms), the carbonyl groups, and the location of the terminal methyl of *sn*-1 chains of POPC. CPT extended from the polar headgroup region up to near the center of the bilayer (z = 0), but there was more overlapping with the carbonyl groups profile (phospholipid/water interface) than with that of the terminal carbons of POPC acyl chains, indicating that the terpenoid was not deeply inserted within the bilayer.

The study of cluster formation of CPT in the membrane showed that CPT molecules are mostly found in the form of monomers, around 66% in POPC and DMPC membranes (Table 2), a result that might seem in contrast to the high hydrophobicity of this molecule. However, observing its molecular structure, the intermolecular interactions would result in steric hindrance, hence the presence of monomers was favored, and only clusters of a few molecules were found. This result excludes CPT clusters as the possible responsible for membrane permeabilization.

**Figure 10:**
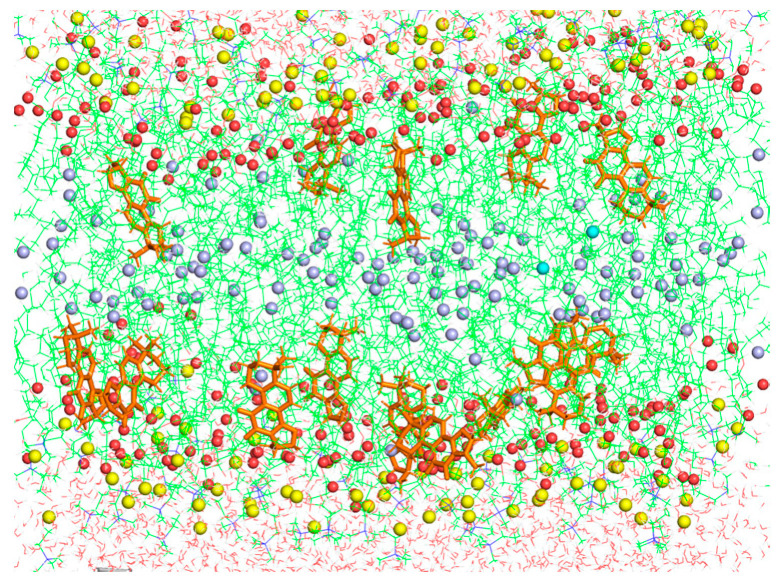
Final snapshot of the simulation box of POPC + CPT membranes. Water molecules are shown in red lines, CPT in orange sticks, POPC atoms in green lines, POPC carbonyl groups in red spheres, POPC methyl terminals of *sn*-1 chains in blue spheres, and phosphorous atoms in yellow spheres.

Figure 10 shows a snapshot corresponding to a final configuration of POPC + CPT membrane simulation. It was observed that clustering of CPT was minimum, and most molecules were monomers or dimers, as shown above. This localization of CPT, closer to the POPC carbonyl groups than to the terminal methyl, was not expected to produce large perturbations in the thermotropic behavior of these systems, as shown above by DSC, due to a minimum effect on lipid packing. However, it will explain the stronger effect on the region of the C=O groups, where significant dehydration was observed by FTIR.

**Figure 11:**
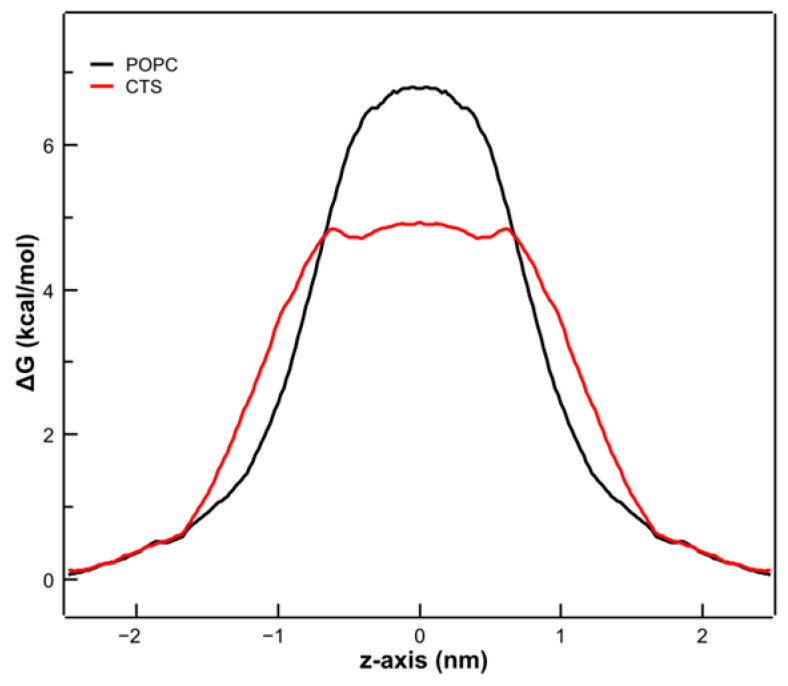
Free energy profiles for a water molecule crossing a POPC membrane (black line) and a POPC + CPT membrane (red line). Profiles are assumed to be symmetric across the bilayer center.

To simulate the effect of CPT on the permeability properties of the membrane to polar substances, the PMF of a water molecule crossing the membrane was determined. The free energy profiles of such a process are shown in Figure 11. The free energy was set to zero in the aqueous phase. In the POPC membrane, the free energy for a water molecule to cross the membrane was about 6.8 ± 1.37 kcal/mol, which is in good agreement with previously reported values ranging from 6.2 to 7 kcal/mol [48,53,54].

It can be observed that in the presence of CPT, the free energy decreased to 4.84 ± 1.22 kcal/mol, clearly indicating that the POPC membrane became more permeable to water in the presence of CPT, making it likely that, by extrapolation, it was also more permeable in general to polar water-soluble compounds. These MD results agree with our experimental data on CF leakage shown above since CF is also a highly polar water-soluble compound, which easily permeates upon the addition of CPT.

## 4. Conclusions

The hydrophobic character of CPT suggests it will exert a strong influence on phospholipid membrane structure and function. We have shown that upon addition to phospholipid liposomes, a fraction of CPT is incorporated into the membrane, resulting in membrane perturbation and content leakage. The finding that CPT presents a limited incorporation into fluid phospholipid membranes, and its tendency to crystallize in water, is an important fact to be taken into consideration relative to its pharmacological use. The effect of CPT mainly occurs at the level of the phospholipid polar headgroups, with little influence on lipid packing, according to FTIR. Thus, CPT increases POPC mobility and dehydrates the polar headgroup region, which could explain membrane permeabilization. The most relevant finding is that CPT membrane effects depend on the membrane phospholipid composition. In particular, the presence of negatively charged phosphatidylglycerol, as a model for cancer and bacterial cells, enhances the activity of CPT, which is important for the explanation of some of its pharmacological actions, since either bacterial or cancer cells normally present negatively charged phospholipids at the outer monolayer. The described effects of CPT on model membranes add to the vast number of biological actions of the terpenoid and enhance its potential, both as an antibacterial agent and for addressing pathogenic tumor cells.

## Figures and Tables

**Table 1:** The effect of CPT on the maximum frequency of the FTIR ν_CH2_ symmetric stretching and ν_CO_ stretching bands of various phosphatidylcholines. The CPTtot/phospholipid molar ratio was 0.1 in all cases. Data correspond to the mean of three independent repetitions ± SD.

Conditions	CH_2_ Symmetric Stretching	C=O Stretching
DMPC 12 °C	2848.3 ± 0.12	1730.9 ± 0.06
DMPC + CPT 12 °C	2849.0 ± 0.05	1733.5 ± 0.09
DMPC 34 °C	2850.1 ± 0.08	1729.8 ± 0.12
DMPC + CPT 34 °C	2851.0 ± 0.04	1731.6 ± 0.06
DPPC 24 °C	2848.6 ± 0.11	1731.4 ± 0.04
DPPC + CPT 24 °C	2849.1 ± 0.03	1733.4 ± 0.03
DPPC 50 °C	2851.1 ± 0.03	1730.7 ± 0.13
DPPC + CPT 50 °C	2851.5 ± 0.03	1732.4 ± 0.10
POPC 25 °C	2848.9 ± 0.08	1726.9 ± 0.05
POPC + CPT 25 °C	2850.3 ± 0.06	1728.7 ± 0.07

**Table 2:** Parameters obtained from MD simulations for pure POPC and DMPC membranes, and after incorporation of CPT.

	POPC	POPC + CPT	DMPC	DMPC + CPT
Area per lipid (nm^2^)	0.63 ± 0.01	0.66 ± 0.02	0.61 ± 0.01	0.64 ± 0.01
Order parameter (S_CD_)	0.14 ± 0.07	0.15 ± 0.07	0.20 ± 0.05	0.193 ± 0.05
Thickness (nm)	4.04 ± 0.07	3.93 ± 0.08	3.81 ± 0.06	3.65 ± 0.07
Diffusion coefficient (10^8^ cm^2^ s^−1^)	5.45 ± 2.19	7.67 ± 2.16	7.3 ± 1.03	11.5 ± 3.2
Cluster (% monomers)	-	66.6 ± 0.9	-	65.6 ± 0.6

## Data Availability

Not applicable.
